# Ultrasonographic Evaluation of the Second Stage of Labor according to the Mode of Delivery: A Prospective Study in Greece

**DOI:** 10.3390/jcm13041068

**Published:** 2024-02-13

**Authors:** Kyriaki Mitta, Ioannis Tsakiridis, Themistoklis Dagklis, Ioannis Kalogiannidis, Apostolos Mamopoulos, Georgios Michos, Andriana Virgiliou, Apostolos Athanasiadis

**Affiliations:** Third Department of Obstetrics and Gynecology, School of Medicine, Faculty of Health Sciences, Aristotle University of Thessaloniki, 54642 Thessaloniki, Greece; kmittb@auth.gr (K.M.); dagklis@auth.gr (T.D.); ikalogia@auth.gr (I.K.); amamop@auth.gr (A.M.); gmichos@auth.gr (G.M.); anvirgiliou@hotmail.gr (A.V.); apathana@auth.gr (A.A.)

**Keywords:** intrapartum ultrasound, ultrasound in labor, second stage, predictor, mode of delivery

## Abstract

Background and Objectives: Accurate diagnosis of labor progress is crucial for making well-informed decisions regarding timely and appropriate interventions to optimize outcomes for both the mother and the fetus. The aim of this study was to assess the progress of the second stage of labor using intrapartum ultrasound. Material and methods: This was a prospective study (December 2022–December 2023) conducted at the Third Department of Obstetrics and Gynecology, School of Medicine, Faculty of Health Sciences, Aristotle University of Thessaloniki, Greece. Maternal–fetal and labor characteristics were recorded, and two ultrasound parameters were measured: the angle of progression (AoP) and the head–perineum distance (HPD). The correlation between the two ultrasonographic values and the maternal–fetal characteristics was investigated. Multinomial regression analysis was also conducted to investigate any potential predictors of the mode of delivery. Results: A total of 82 women at the second stage of labor were clinically and sonographically assessed. The mean duration of the second stage of labor differed between vaginal and cesarean deliveries (65.3 vs. 160 min; *p*-value < 0.001) and between cesarean and operative vaginal deliveries (160 vs. 88.6 min; *p*-value = 0.015). The occiput anterior position was associated with an increased likelihood of vaginal delivery (OR: 24.167; 95% CI: 3.8–152.5; *p*-value < 0.001). No significant differences were identified in the AoP among the three different modes of delivery (vaginal: 145.7° vs. operative vaginal: 139.9° vs. cesarean: 132.1°; *p*-value = 0.289). The mean HPD differed significantly between vaginal and cesarean deliveries (28.6 vs. 41.4 mm; *p*-value < 0.001) and between cesarean and operative vaginal deliveries (41.4 vs. 26.9 mm; *p*-value = 0.002); it was correlated significantly with maternal BMI (r = 0.268; *p*-value = 0.024) and the duration of the second stage of labor (r = 0.256; *p*-value = 0.031). Low parity (OR: 12.024; 95% CI: 6.320–22.876; *p*-value < 0.001) and high HPD (OR: 1.23; 95% CI: 1.05–1.43; *p*-value = 0.007) were found to be significant predictors of cesarean delivery. Conclusions: The use of intrapartum ultrasound as an adjunctive technique to the standard clinical evaluation may enhance the diagnostic approach to an abnormal labor progress and predict the need for operative vaginal or cesarean delivery.

## 1. Introduction

For women undergoing labor without neuraxial anesthesia, the second stage usually lasts fewer than three hours for nulliparous individuals and fewer than two hours for multiparous ones; in cases with neuraxial anesthesia, this may be extended by one hour [1,2]. If the duration exceeds these timeframes, it is defined as prolonged; various factors, including fetal size and position, maternal pelvic shape, expulsive efforts, maternal age, obstetric history, and comorbidities such as hypertension or diabetes, may affect the length of the second stage of labor [3].

Traditionally, the assessment and management of the progress of labor are based on clinical assessment [4,5,6]. The diagnosis of labor arrest and decisions regarding the timing or type of intervention rely mostly on digital evaluation of cervical dilatation and fetal head station/position [7,8]. However, clinical examination of the head station and position may be inaccurate and subjective, especially when caput succedaneum impairs palpation of the sutures and fontanels. The use of ultrasound has been suggested as a valuable tool in labor management; numerous studies have highlighted the superiority of ultrasound examination compared to clinical assessment in diagnosing fetal head position and station and predicting labor arrest [9,10,11,12,13,14,15]. Ultrasound examinations can, to some degree, differentiate between women likely to have a spontaneous vaginal delivery and those who may require an operative vaginal delivery [10,15,16,17,18,19,20]. Intrapartum ultrasound can be conducted through a transabdominal approach, primarily for determining head and spine position, or a transperineal approach to assess the head station and position at lower stations; the most common quantitative sonographic parameters are the angle of progression (AoP) and the head–perineum distance (HPD) [21].

Currently, there is no consensus on the appropriate timing of intrapartum ultrasound use, which specific parameters should be obtained, or how the sonographic findings should be integrated into clinical practice to enhance patient management. Nevertheless, achieving a precise diagnosis of labor progress is essential for making informed decisions about interventions while also ensuring that appropriate measures are taken at the right time to optimize outcomes for both the mother and the fetus.

Thus, the aim of this study was to evaluate the use of intrapartum ultrasound during the second stage of labor.

## 2. Material and Methods

### 2.1. Study Design/Parameters

This was a prospective study (December 2022–December 2023) conducted at the Third Department of Obstetrics and Gynecology, School of Medicine, Faculty of Health Sciences, Aristotle University of Thessaloniki, Greece. This study was approved by the Ethics Committee of the Aristotle University of Thessaloniki (3–13 December 2022). Informed consent was obtained before the procedure, and no incentives were provided for participation in the study.

Maternal (age, BMI, parity), fetal/neonatal (fetal head position, birth weight), and labor characteristics (total duration of labor, duration of the second stage of labor, epidural and oxytocin use, spontaneous onset of labor or induction of labor) were recorded, and the AoP and the HPD were measured.

In cases of induction of labor, the Βishop score was calculated to choose the method of induction; if the Bishop score was favorable (≥6), rupture of membranes with or without oxytocin was the preferrable method of labor induction, whereas when the Bishop score was unfavorable (<6), cervical ripening agents were used first [22]. Induction of labor was performed according to the guidelines of the Hellenic Society of Obstetrics and Gynecology. Regarding cervical ripening agents, either PGE2 (dinoprostone) or PGE1 (misoprostol) was administered if Bishop score was unfavorable; a PGE2 suppository of 3 mg was administered vaginally, with a minimum safe time interval between prostaglandin administration and oxytocin initiation of at least 6 h, or 25–50 mcg of PGE1 was administered as an initial dose for cervical ripening and induction of labor, and oxytocin was administered at least 4 h after the last misoprostol dose. If there was inadequate cervical change with minimal uterine activity after one dose of either intracervical dinoprostone or misoprostol, a second dose was given 6 h later [22].

Operative vaginal delivery (OVD) was attempted when the fetal head station was below +1 level, the AoP was more than 130° (+1), or the HPD was less than 35 mm (mid-cavity), or if the second stage of labor lasted for more than 4 h in primiparous patients with epidural, more than 3 h in primiparous patients without epidural, more than 3 h in multiparous patients with epidural, or more than 2 h in multiparous patients without epidural [23].

### 2.2. Technique

The sonographic assessment of fetal head station is performed by transperineal ultrasound in the midsagittal or axial plane. The probe is placed between the two labia majora, at the level of the fourchette, with the woman in a semi-recumbent position [21]. The AoP is the angle between the long axis of the pubic bone and a line drawn from the lowest edge of the pubis tangential to the deepest bony part of the fetal skull. It is an accurate and reproducible parameter for the assessment of fetal head descent [20,24]. An angle of progression of 106° has been found to correspond to fetal head station 0 (zero) [20]. The HPD is measured by placing the probe between the labia majora, and the soft tissue is compressed completely against the pubic bone. The transducer should be angled until the skull contour is as clear as possible, indicating that the ultrasound beam is perpendicular to the fetal skull. HPD is measured in a frontal transperineal scan as the shortest distance from the outer bony limit of the fetal skull to the perineum [21,25].

### 2.3. Statistical Analysis

Ordinal or qualitative data were described as *n* (%), and quantitative data as the mean (SD). A one-way ANOVA test was conducted to compare the means of the quantitative variables. Post hoc analysis was conducted for significant values. The association between the mode of delivery (cesarean section—CS, vaginal delivery—VD, OVD) and the head position (occiput anterior vs. occiput posterior), as assessed by transabdominal ultrasound, was investigated by means of multinomial regression analysis. The correlation between the two ultrasonographic values (AoP, HPD) and the different maternal–fetal characteristics was investigated with Pearson’s (r) correlation coefficient. Additionally, multinomial regression analysis was conducted to investigate any potential predictors of the mode of delivery and any association between epidural, oxytocin use, and the onset of labor (spontaneous vs. induced) with the mode of delivery (VD, OVD, CS). The means of the ultrasonographic parameters (AoP, HPD) were compared between the parturients with normal duration of the second stage and those with a prolonged one using Student’s t-test. The level of statistical significance was defined as *p* = 0.05. The statistical package IBM SPSS Statistics 29.0 was used.

## 3. Results

A total of 82 women at the second stage of labor were assessed both clinically and sonographically. The mean maternal age was 28 years (SD: 6.5), the mean gestational age was 39 weeks (SD: 1.4), the mean parity was 1.3 (IQR: 0.7), and the mean body mass index (BMI) was 29.6 kg/m^2^ (SD: 5). The onset of labor was spontaneous in 35 cases (42.7%), while 47 (57.3%) women underwent induction of labor. During labor, 41 women received epidurals (50%) and 59 oxytocin (71.9%). Moreover, 60 women (73.2%) delivered via VD, 11 (13.4%) underwent OVD, and 11 (13.4%) delivered via CS. The mean total duration of labor was 10.5 h (SD: 6.2), whereas the mean duration of the second stage of labor was 81 min (SD: 67). The mean birth weight was 3252 g (SD: 492).

In total, out of 32 women who underwent induction of labor and delivered vaginally, 15 had a favorable Bishop score (≥6), whereas 17 had an unfavorable Bishop score (<6). Out of six women who underwent induction of labor and delivered via CS, all of them had unfavorable Bishop scores before induction of labor. Out of nine parturients who underwent induction and delivered via OVD, all had unfavorable Bishop scores. In total, out of 47 inductions of labor, 15 had favorable Bishop scores, whereas 32 had unfavorable ones. Following the analyses, we found a significant difference in the Bishop scores between the VD and CS (*p*-value < 0.001) groups and between VD and OVD (*p*-value = 0.012), but not between CS and OVD (*p*-value = 0.288).

Maternal and fetal characteristics were analyzed according to the three modes of delivery (VD, OVD, CS) (Table 1). The mean total duration of labor differed between VD and CS (8.8 vs. 16.1 h, *p*-value < 0.001) and between VD and OVD (8.8 vs. 14.2 h, *p*-value = 0.011); however, it did not differ significantly between CS and OVD. Moreover, the mean duration of the second stage of labor differed between VD and CS (65.3 vs. 160 min, *p*-value < 0.001) and OVD and CS (88.6 vs. 160 min, *p*-value = 0.015). The mean birth weight differed significantly between neonates born vaginally and those via CS (3161 vs. 3572 g, *p*-value = 0.026).

**Figure 1:**
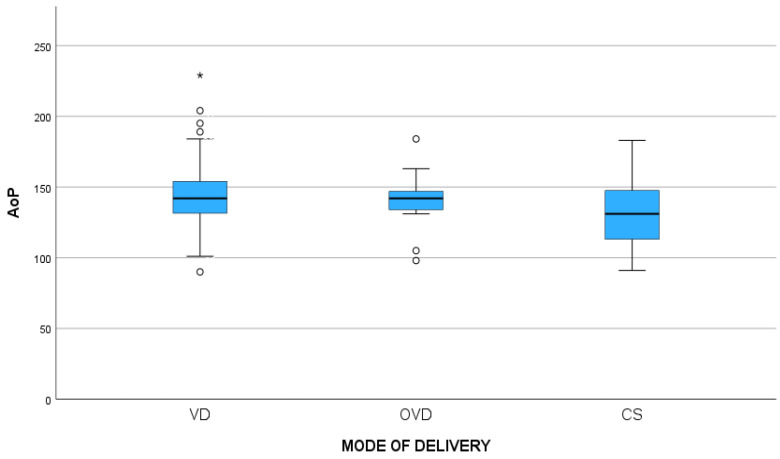
Comparisons of means of angle of progression (AoP) between different modes of delivery (*p*-value = 0.289). The dots “°” in the figure are the outliers; the values that fall above or below the expected variation and the asterisk “*” is the extreme outlier.

**Figure 2:**
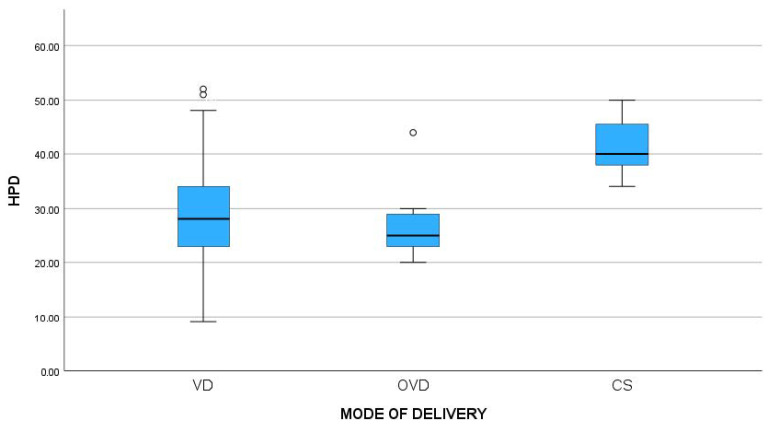
Comparisons of means of head–perineum distance (HPD) between different modes of delivery (*p*-value < 0.001). The dots “°” in the figure are the outliers; the values that fall above or below the expected variation.

The association between the mode of delivery and fetal head position (occiput posterior vs. anterior) was also assessed. The occiput anterior position was associated with increased odds of VD compared to CS (OR: 24.167, 95% CI: 3.8–152.5, *p*-value < 0.001) (Table 2). The mean AoP did not differ significantly among the three groups (145.7° for VD vs. 139.9° for OVD vs. 132.1° for CS, *p*-value = 0.289). On the contrary, the mean HPD was significantly higher in the CS cases (28.6 vs. 26.9 vs. 41.4, *p*-value < 0.001) (Table 3). Moreover, post hoc analysis revealed that the mean HPD differed between VD and CS (*p*-value < 0.001) and CS and OVD (*p*-value = 0.002), but not between VD and OVD (Figure 1 and Figure 2).

The correlation of AoP with maternal–fetal characteristics was investigated, and we identified a negative association between parity and AoP (Pearson correlation coefficient r = −0.221, *p*-value = 0.047). In addition, a positive association was identified between HPD and BMI (Pearson correlation coefficient r = 0.268, *p*-value = 0.024), as well as the duration of the second stage of labor (Pearson correlation coefficient r = 0.256, *p*-value = 0.031) (Table 4).

Multinomial regression analysis was conducted to investigate any potential predictors of the mode of delivery. Low parity (OR: 12.024, 95% CI: 6.320–22.876, *p*-value < 0.001) and increased HPD (OR: 1.23, 95% CI: 1.05–1.43, *p*-value = 0.007) were found to be significant predictors of CS. The total duration of labor was found to be a significant predictor of OVD (OR: 1.24, 95% CI: 1.05–1.46, *p*-value = 0.007) (Table 5).

Multinomial regression analysis was conducted to investigate any associations between epidural (yes/no), oxytocin (yes/no), or onset of labor (spontaneous/induction of labor) and the mode of delivery (VD, OVD, or CS). Neither epidural nor oxytocin, nor onset of labor could predict the mode of delivery (Table 6).

**Figure 3:**
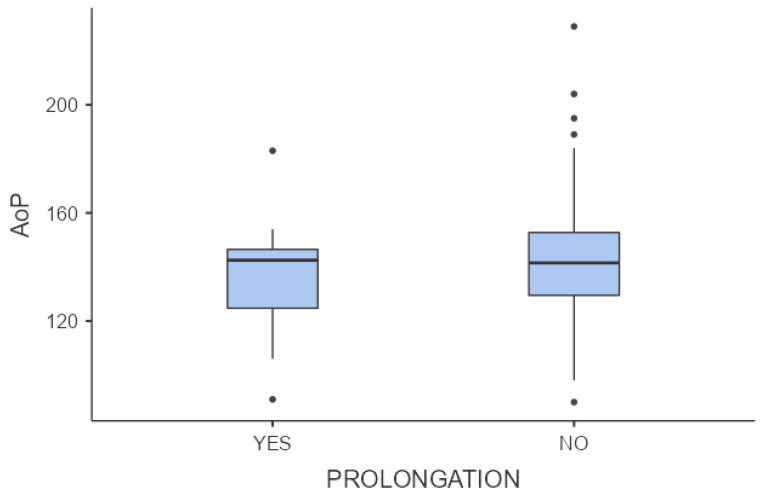
Comparisons of means of angle of progression (AoP) between normal and prolonged second stage of labor (*p*-value = 0.485). The dots “°” in the figure are the outliers; the values that fall above or below the expected variation.

**Figure 4:**
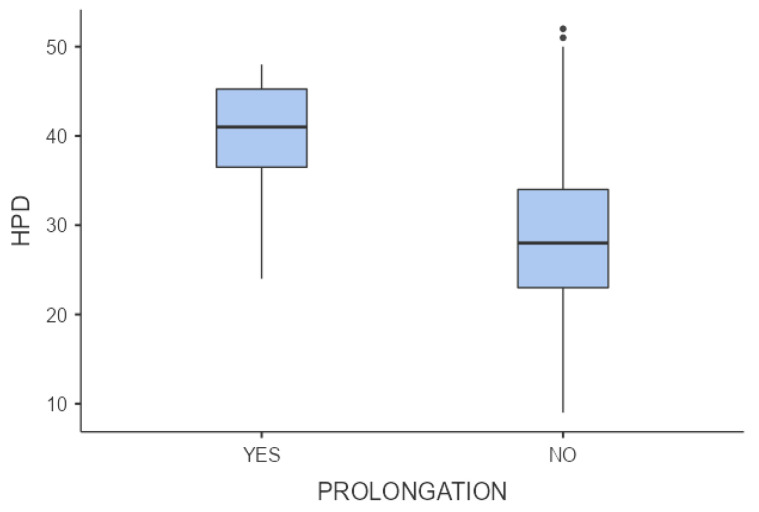
Comparisons of means of head–perineum distance (HPD) between normal and prolonged second stage of labor (*p*-value = 0.005).

In 11 (13.4%) cases, there was prolongation of the second stage, according to the criteria defined by Zhang et al. [6]. In 2 of these 11 (18.2%) cases, there was an occiput posterior head position; a successful OVD was performed in 1 case, whereas a CS was performed in the other one. The mean AoP did not differ significantly between the groups with prolonged and normal second stages of labor (136.8º vs. 143.8°, *p*-value = 0.458), whereas the HPD differed significantly between the two groups (39.6 vs. 29.1, *p* = 0.005) (Figure 3 and Figure 4).

## 4. Discussion

The main findings of this study were (i) the mean total duration of labor was lower in the VD compared to the CS group; (ii) the mean duration of the second stage of labor was lower in the VD and OVD compared to the CS group; (iii) the occiput anterior position was associated with an increased likelihood of VD; (iv) the mean HPD was lower in the VD compared to the CS group and was associated with increasing maternal BMI and a prolonged second stage of labor; (v) low parity and high HPD were found to be significant predictors of CS; and (vi) increased total duration of labor was a significant predictor of OVD.

A study examining the mode of delivery and outcomes according to birth weight revealed that birth weight is a significant determinant of the mode of delivery; CS rates were increased with increasing birth weight [26], which was in accordance with the findings of our study. Additionally, published data support that longer durations of both the first and the second stages of labor have been associated with higher odds of OVD [27]. Our study revealed a significant difference in the total duration of labor and the duration of the second stage of labor between the delivery modes. Moreover, according to the literature, only 34% of cases with occiput posterior head position do not require any operative intervention, whereas 82% of cases with occiput anterior head position do not require any operative intervention [28,29], which is in agreement with the findings of our study.

Regarding the prediction of the mode of delivery following the measurement of the AoP, the current literature supports that this sonographic parameter can predict the duration of labor, the progress, and the mode of delivery [30]; a larger angle at the beginning of the second stage of labor has been significantly associated with shorter time to delivery [31]. More specifically, one study suggested that an AoP ≥ 113° at the second stage of labor was associated with 90% probability of VD [31]. In another study, the odds of OVD were 2.6 times higher for women with an AoP < 153°, and the odds of CS were almost six times higher when compared with women with AoP ≥ 153° (aOR: 5.8, 95% CI: 1.2–28.3, *p* = 0.03) [32]. According to the results of our study, the mean AoP differed among the three groups (VD, OVD, CS), but not significantly; therefore, it could not predict the mode of delivery in our sample. This could be attributed to the small sample and/or to the fact that a single value of AoP was measured at the beginning of the second stage, without serial measurements unless there was a prolonged second stage.

Regarding HPD, according to published data, VD has been associated with a lower HDP in comparison with those who required OVD or CS (33.2 mm vs. 40.1, *p*-value = 0.001) [29]; this is in accordance to the findings of our study. High parity, along with low HPD, have been previously identified as significant predictors of VD, which is also in agreement with our findings [17]. In case of a prolonged second stage of labor, the literature supports that both the AoP and the HPD can predict VD [29]. We found that HPD was significantly higher in cases of prolonged second stages of labor. It has been shown that multiparous women maintain a higher station for a longer time before delivery, but often proceed rapidly to delivery once full dilation is reached [33]. Moreover, we found that AoP was correlated negatively with parity; the first measurement of AoP was lower in multiparous than nulliparous patients, despite the shorter durations of their second stages.

This study’s main strength lies in its comprehensive approach, integrating both ultrasound and clinical parameters to assess labor progression, particularly during the second stage. Moreover, its prospective nature adds to the strengths of the study. However, it is essential to acknowledge the study’s limitations, especially the limited sample size. Another limitation includes the single measurement of the AoP and HDP during the second stage of labor; serial measurements were conducted only in cases of prolonged second stage duration.

## 5. Conclusions

According to the results of this study, the use of intrapartum ultrasound as an adjunctive technique to the standard clinical evaluation may predict the need for OVD or CS. The optimal mode of delivery remains a prominent concern in modern obstetrics. Over recent years, there has been a progressive increase in the rates of CS, surpassing the recommended limit set by medical societies. Obstetricians often face a challenge, as they lack the requisite technology to assist in determining the appropriateness of a CS based on intrapartum conditions. Integrating intrapartum ultrasound in the assessment of the progress of labor, along with all the clinical parameters, may enhance the diagnostic approach to an abnormal labor progress.

## Figures and Tables

**Table 1:** Baseline characteristics according to the mode of delivery.

Characteristics	Mode of Delivery			
	Vaginal Delivery (VD) *n* = 60 (73.2%)	Operative Vaginal Delivery (OVD) *n* = 11 (13.4%)	Cesarean Section (CS) *n* = 11 (13.4%)	*p*-Value
**Maternal age (years)**	27.7 (6.9)	29.1 (4.3)	28.9 (6.7)	0.736
**Maternal BMI (kg/m^2^)**	29.5 (5.3)	29.6 (4.4)	30.4 (4.6)	0.876
**Gestational age at delivery (weeks)**	39 (1.4)	39.3 (1.2)	39.4 (0.9)	0.053
**Parity**	1.3 (0.6)	1.3 (1.2)	1 (0.3)	0.545
**Birth weight (g)**	3161 (508)	3429 (249)	3572 (416)	0.015
**Duration of second stage of labor (minutes)**	65.3 (57.6)	88.6 (67.6)	160 (61.2)	<0.001
**Total duration of labor (hours)**	8.8 (4.9)	14.2 (5.8)	16.1 (8.3)	<0.001

Data are given as *n* (%), mean (SD) for parametric values. One-way ANOVA was used for parametric values; SD: standard deviation; IQR: interquartile range; BMI: body mass index.

**Table 2:** Mode of delivery according to fetal head position.

Mode of Delivery	Head Position		ORs	95% CI	*p*-Value
	*Occiput Anterior*	*Occiput Posterior*			
*Vaginal delivery*	58 (96.7%)	2 (3.3%)	24.167	3.8–152.5	<0.001
*Operative vaginal delivery*	10 (90.9%)	1 (9.1%)	8.333	0.7–89.4	0.080
*Cesarean section*	6 (54.5%)	5 (45.5%)	Reference		

Data are given as n (%), multinomial regression analysis was used. ORs: odds ratios, 95% CI: 95% confidence interval.

**Table 3:** Ultrasonographic characteristics at the second stage of labor, according to the mode of delivery.

Ultrasonographic Characteristics	Mode of Delivery			*p*-Value
	Vaginal Delivery	Operative Vaginal Delivery	Cesarean Section	
**HPD (mm)**	28.6 (10.1)	26.9 (6.84)	41.4 (5.3)	<0.001
**AoP (°)**	145.7 (24.9)	139.9 (23.8)	132.1 (26.2)	0.289

One-way ANOVA, AoP: angle of progression, HPD: head–perineum distance.

**Table 4:** Correlation of head–perineum distance (HPD) with maternal–fetal characteristics.

Fetal–Maternal Characteristics	HPD		AoP	
	Pearson r Correlation Coefficient	*p*-Value	Pearson r Correlation Coefficient	*p*-Value
Age (years)	0.084	0.484	0.045	0.686
Gestational age (weeks)	0.128	0.288	−0.187	0.093
Parity	0.226	0.060	−0.221	0.047
BMI (kg/m^2^)	0.268	0.024	0.011	0.921
Birth weight (grams)	−0.002	0.985	0.013	0.909
Duration of the second stage of labor (min)	0.256	0.031	−0.186	0.095
Total duration of labor (hours)	0.098	0.418	0.012	0.918

Pearson’s correlation coefficient r was used for the parametric values.

**Table 5:** Predictors of mode of delivery.

Mode of Delivery	Predictor	ORs	95% CI	*p*-Value
Cesarean section vs. Vaginal delivery (Ref)	Age	1.2	0.003–0.003	0.451
	Gestational age	0.68	0.42–1.12	0.133
	Low parity	12.024	6.320–22.876	<0.001
	BMI	0.903	0.700–1.164	0.432
	Birth weight	1	0.99–1	0.079
	Duration of the second stage of labor	1	0.99–1.03	0.208
	Total duration of labor	1.1	0.94–1.34	0.178
	High AoP	1	0.9–1.06	0.655
	High HPD	1.23	1.05–1.43	0.007
Operative vaginal delivery vs. Vaginal delivery (Ref)	Age	1.06	0.942–1.202	0.312
	Gestational age	1.29	0.96–1.73	0.086
	Low parity	1.99	0.55–7.166	0.292
	BMI	1.04	0.86–1.2	0.645
	Birth weight	1	0.99–1	0.292
	Duration of the second stage of labor	1	0.98–1.01	0.821
	Total duration of labor	1.24	1.05–1.46	0.009
	High AoP	0.98	0.94–1.02	0.421
	High HPD	0.91	0.78–1.06	0.244

Vaginal delivery: reference (Ref), ORs: odds ratios, 95% CI: 95% confidence interval, BMI: body mass index, multinomial regression analysis.

**Table 6:** Association of oxytocin, epidural, and spontaneous onset of labor versus induction of labor with the mode of delivery.

Mode of Delivery	Labor Characteristics		ORs	95% CI	*p*-Value
**Cesarean section**	**Oxytocin**		4.603	0.547–38.76	0.160
	*Yes*	10 (16.9%)			
	*No*	1 (4.3%)			
	**Epidural**		1.121	0.286–4.390	0.870
	*Yes*	6 (14.6%)			
	*No*	5 (12.2%)			
	**Onset of labor**		1.051	0.248–4.124	0.943
	*Spontaneous*	5 (14.3%)			
	*Induction*	6 (12.8%)			
**Operative Vaginal Delivery**	**Oxytocin**		1.168	0.267–5.107	0.837
	*Yes*	8 (13.6%)			
	*No*	3 (13.0%)			
	**Epidural**		0.572	0.147–2.233	0.422
	*Yes*	5 (12.2%)			
	*No*	6 (14.6%)			
	**Onset of labor**		0.219	0.041–1.159	0.074
	*Spontaneous*	2 (5.7%)			
	*Induction*	9 (19.1%)			
**Vaginal Delivery**	**Oxytocin**		*Reference*		
	*Yes*	41 (69.5%)			
	*No*	19 (82.6%)			
	**Epidural**				
	*Yes*	30 (73.2%)			
	*No*	30 (73.2%)			
	**Onset of labor**				
	*Spontaneous*	28 (80.0%)			
	*Induction*	32 (68.1%)			

ORs: odds ratios, 95% CI: 95% confidence interval, vaginal delivery: reference; multinomial regression analysis was used.

## Data Availability

Data are available upon request.
